# The genome sequence of the satellite, *Eupsilia transversa* (Hufnagel, 1766)

**DOI:** 10.12688/wellcomeopenres.18105.1

**Published:** 2022-10-19

**Authors:** Liam Crowley, David Lees, University of Oxford and Wytham Woods Genome Acquisition Lab, Natural History Museum Genome Acquisition Lab, Darwin Tree of Life Barcoding collective, Wellcome Sanger Institute Tree of Life programme, Wellcome Sanger Institute Scientific Operations: DNA Pipelines collective, Tree of Life Core Informatics collective, Finley Hutchinson, Darwin Tree of Life Consortium

**Affiliations:** 1 Department of Zoology, University of Oxford, Oxford, Oxfordshire, UK; 2 Natural History Museum, London, UK; 3 Independent reseacher, Reading, Berkshire, UK

**Keywords:** Eupsilia transversa, the satellite, genome sequence, chromosomal, Lepidoptera

## Abstract

We present a genome assembly from an individual female *Eupsilia transversa* (the satellite; Arthropoda; Insecta; Lepidoptera; Noctuidae). The genome sequence is 467 megabases in span. The entire assembly (100%) is scaffolded into 32 chromosomal pseudomolecules with the W and Z sex chromosomes assembled. The complete mitochondrial genome was also assembled and is 15.5 kilobases in length. Gene annotation of this assembly on Ensembl has identified 18,065 protein coding genes.

## Species taxonomy

Eukaryota; Metazoa; Ecdysozoa; Arthropoda; Hexapoda; Insecta; Pterygota; Neoptera; Endopterygota; Lepidoptera; Glossata; Ditrysia; Noctuoidea; Noctuidae; Ipimorphinae; *Eupsilia*; *Eupsilia transversa* (Hufnagel, 1766) (NCBI:txid116130).

## Background

The satellite, *Eupsilia transversa* (Hufnagel, 1766), is a medium-sized Noctuid moth, typically with a red-brown ground colour and a white to orange, reniform stigma on each wing. Each stigma has a small, diagnostic “satellite” dot on either side of it, giving the moth its vernacular name. The species shows a large degree of colour variation throughout its range and several aberrations have been named, mainly based on ground colour and colour of the stigmata ( Heath & Emmett, 1983).

The Satellite is found throughout Eurasia ( Heath & Emmett, 1983); in Britain it is widespread and common throughout, and is also widespread but more localised in Ireland. They occur in one generation, emerging in late September or October and overwintering, flying on milder nights until late April ( Waring & Townsend, 2017).

The larvae, which can be found between April and July in a variety of habitats, are omnivorous, feeding on a wide range of trees as shrubs at first as well as other larvae and aphids when they are larger. The larvae themselves are brown to blue-black with orange or yellow dorsal and subdorsal lines on the first and last body segments, as well as faint dorsal and subdorsal lines along the other segments. They often show white blotches and dashes along the subspiracular line. The larvae feed at night and hide in spun leaves by day, before forming a cocoon on the ground ( Henwood & Sterling, 2020).

The adults can be attracted to light traps, but are more frequently encountered at ‘sugar’ (strong, sweet solutions painted onto tree trunks, fence posts, etc.). The satellite has been recorded feeding on ivy blossom, birch sap and sallow, and they have also been noted feeding on berries including those of Guelder-rose ( Gordon, 1913; Waring & Townsend, 2017).

## Genome sequence report

**Figure 1.**
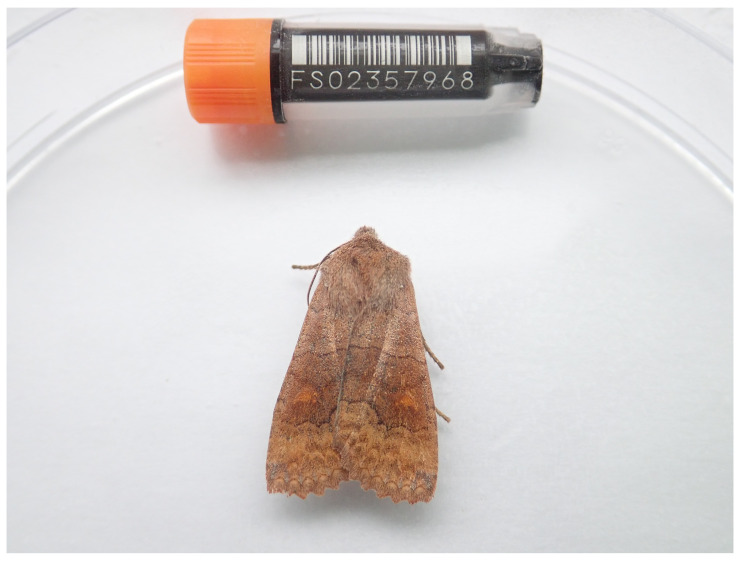
Image of the female *Eupsilia transversa* specimen (ilEupTran1) taken prior to preservation and processing.

The genome was sequenced from a single female *E. transversa* collected from Wytham Woods, Berkshire, UK ( Figure 1). A total of 29-fold coverage in Pacific Biosciences single-molecule HiFi long reads and 53-fold coverage in 10X Genomics read clouds were generated. Primary assembly contigs were scaffolded with chromosome conformation Hi-C data. Manual assembly curation corrected 12 misjoins, reducing the scaffold number by 27.27%, and increasing the scaffold N50 by 3.29%.

**Figure 2.**
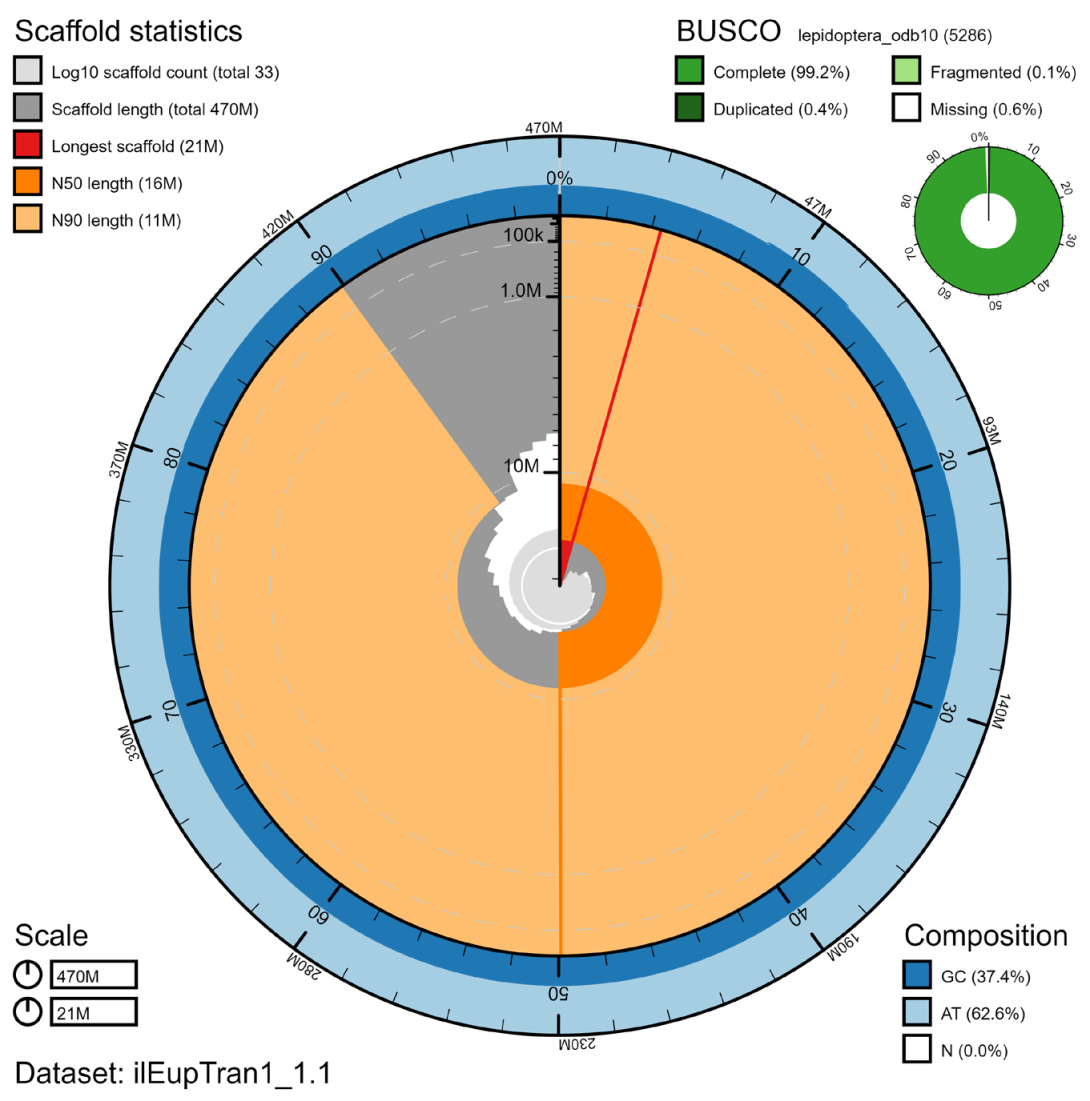
Genome assembly of *Eupsilia transversa*, ilEupTran1.1: metrics. The BlobToolKit Snailplot shows N50 metrics and BUSCO gene completeness. The main plot is divided into 1,000 size-ordered bins around the circumference with each bin representing 0.1% of the 466,922,763 bp assembly. The distribution of chromosome lengths is shown in dark grey with the plot radius scaled to the longest chromosome present in the assembly (20,783,224 bp, shown in red). Orange and pale-orange arcs show the N50 and N90 chromosome lengths (15,778,023 and 10,879,162 bp), respectively. The pale grey spiral shows the cumulative chromosome count on a log scale with white scale lines showing successive orders of magnitude. The blue and pale-blue area around the outside of the plot shows the distribution of GC, AT and N percentages in the same bins as the inner plot. A summary of complete, fragmented, duplicated and missing BUSCO genes in the lepidoptera_odb10 set is shown in the top right. An interactive version of this figure is available at https://blobtoolkit.genomehubs.org/view/ilEupTran1.1/dataset/ilEupTran1_1.1/snail.

The final assembly has a total length of 467 Mb in 32 sequence scaffolds with a scaffold N50 of 15.8 Mb ( Table 1). The entire assembly sequence (100%) was assigned to 32 chromosomal-level scaffolds, representing 30 autosomes (numbered by sequence length) and the W and Z sex chromosome ( Figure 2– Figure 5; Table 2).

**Table 1.** Genome data for *Eupsilia transversa*, ilEupTran1.1.

*Project accession data*	
Assembly identifier	ilEupTran1.1
Species	*Eupsilia transversa*
Specimen	ilEupTran1 (genome assembly, Hi-C); ilEupTran2 (RNA-Seq)
NCBI taxonomy ID	116130
BioProject	PRJEB46318
BioSample ID	SAMEA8563699
Isolate information	Female (ilEupTran1); abdomen/ thorax tissue (genome assembly), head tissue (Hi-C). Unknown sex (ilEupTran2); thorax tissue (RNA-Seq).
*Raw data accessions*	
PacificBiosciences SEQUEL II	ERR6808002;ERR6939241
10X Genomics Illumina	ERR6688520-ERR6688523
Hi-C Illumina	ERR6688519
PolyA RNA-Seq Illumina	ERR9435005
*Genome assembly*	
Assembly accession	GCA_914767815.1
*Accession of alternate * *haplotype*	GCA_914767805.1
Span (Mb)	467
Number of contigs	51
Contig N50 length (Mb)	15.3
Number of scaffolds	32
Scaffold N50 length (Mb)	15.8
Longest scaffold (Mb)	19.9
BUSCO * genome score	C:99.2%[S:98.8%,D:0.4%],F:0.1%, M:0.6%,n:5,286

*BUSCO scores based on the lepidoptera_odb10 BUSCO set using v5.3.2. C= complete [S= single copy, D=duplicated], F=fragmented, M=missing, n=number of orthologues in comparison. A full set of BUSCO scores is available at https://blobtoolkit.genomehubs.org/view/ilEupTran1.1/dataset/ilEupTran1_1.1/busco.

**Figure 3.**
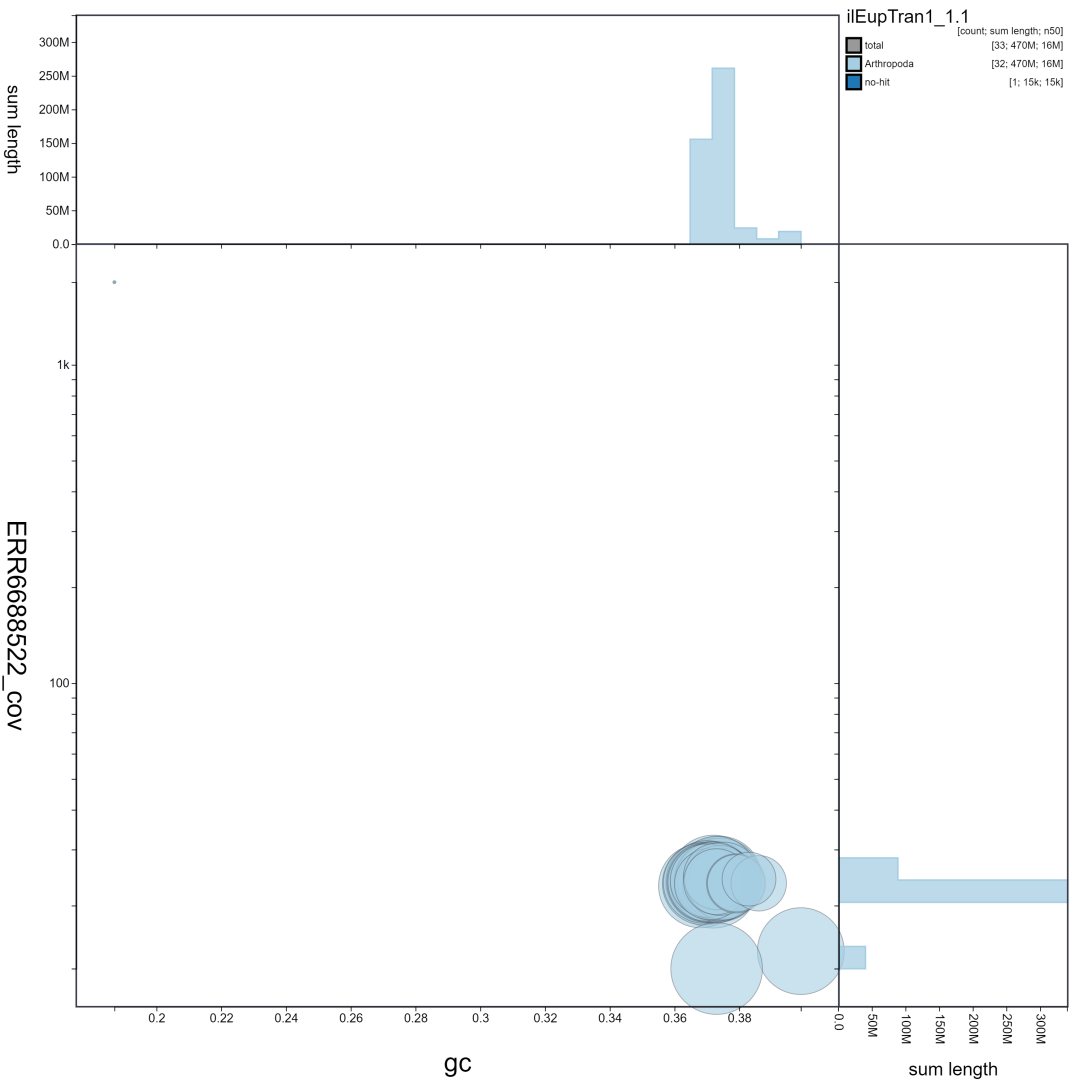
Genome assembly of *Eupsilia transversa*, ilEupTran1.1: GC coverage. BlobToolKit GC-coverage plot. Scaffolds are coloured by phylum. Circles are sized in proportion to scaffold length. Histograms show the distribution of scaffold length sum along each axis. An interactive version of this figure is available at https://blobtoolkit.genomehubs.org/view/ilEupTran1.1/dataset/ilEupTran1_1.1/blob.

**Figure 4.**
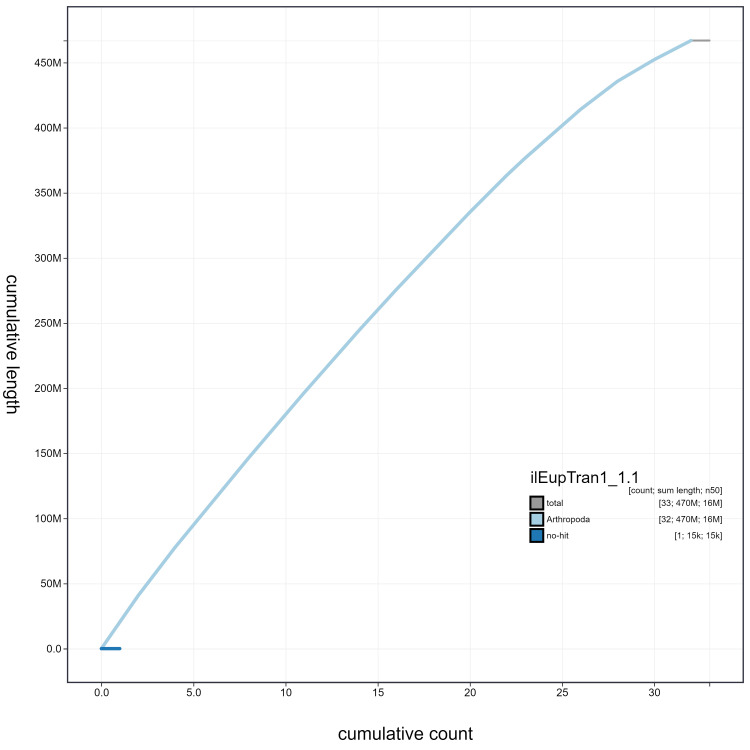
Genome assembly of *Eupsilia transversa*, ilEupTran1.1: cumulative sequence. BlobToolKit cumulative sequence plot. The grey line shows cumulative length for all scaffolds. Coloured lines show cumulative lengths of scaffolds assigned to each phylum using the buscogenes taxrule. An interactive version of this figure is available at https://blobtoolkit.genomehubs.org/view/ilEupTran1.1/dataset/ilEupTran1_1.1/cumulative.

**Figure 5.**
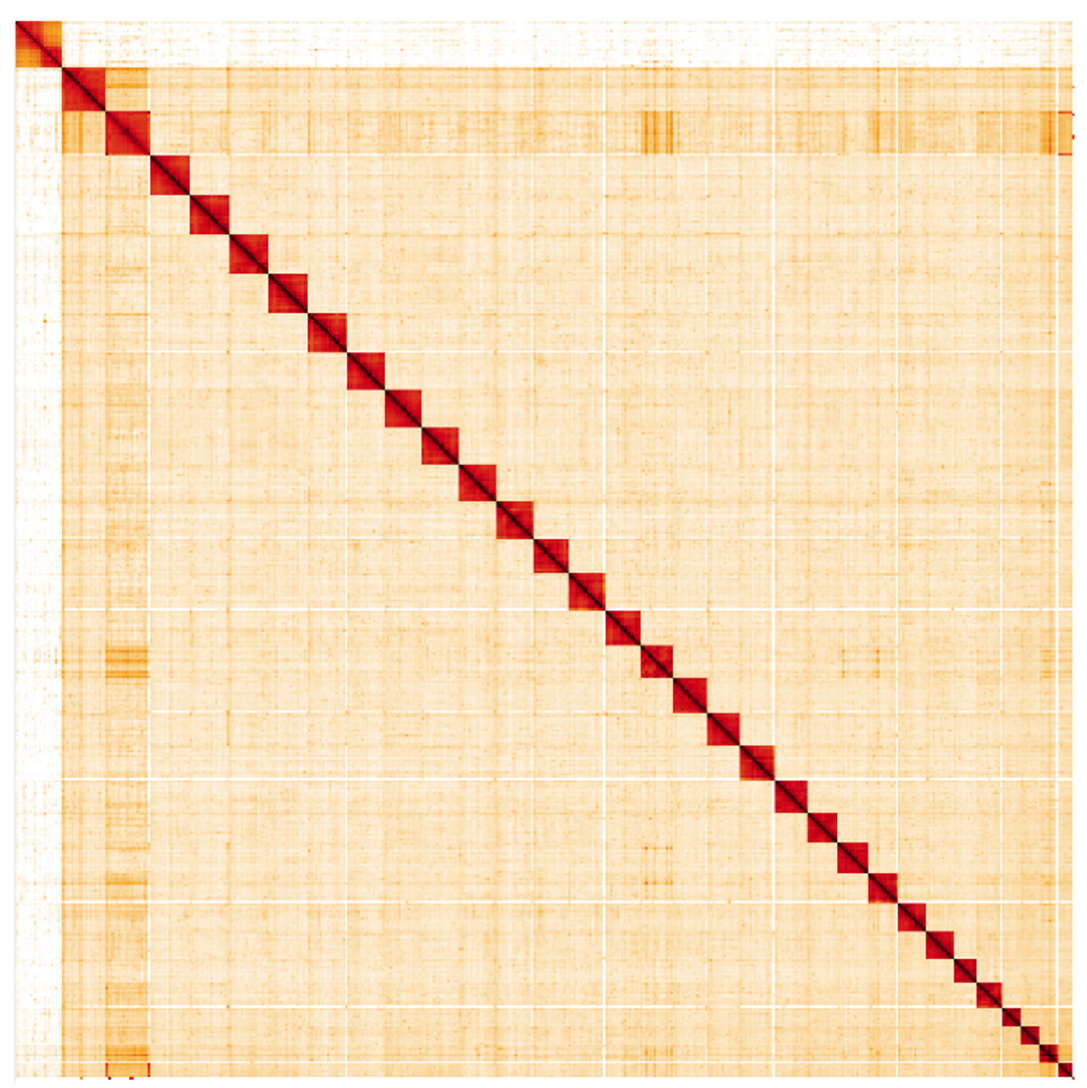
Genome assembly of *Eupsilia transversa*, ilEupTran1.1: Hi-C contact map. Hi-C contact map of the ilEupTran1.1 assembly, visualised in HiGlass. Chromosomes are arranged in size order from left to right and top to bottom. The interactive Hi-C map can be viewed at https://genome-note-higlass.tol.sanger.ac.uk/l/?d=DwMewjHPQq2H4jyHyzVEFg.

**Table 2.** Chromosomal pseudomolecules in the genome assembly of *Eupsilia transversa*, ilEupTran1.1.

INSDC accession	Chromosome	Size (Mb)	GC%
OU611872.1	1	19.97	37.2
OU611874.1	2	18.35	37.2
OU611875.1	3	17.37	36.8
OU611876.1	4	17.35	37.4
OU611877.1	5	17.26	37.4
OU611878.1	6	16.83	36.9
OU611879.1	7	16.74	37
OU611880.1	8	16.56	37.2
OU611881.1	9	16.53	37.3
OU611882.1	10	16.33	37
OU611883.1	11	16.13	36.9
OU611884.1	12	15.78	37
OU611885.1	13	15.71	37.1
OU611886.1	14	15.28	37.2
OU611887.1	15	15.03	37.3
OU611888.1	16	15.01	37
OU611889.1	17	14.75	37.3
OU611890.1	18	14.71	37.2
OU611891.1	19	14.43	37.5
OU611892.1	20	13.89	37.4
OU611893.1	21	13.25	37
OU611894.1	22	12.61	37.5
OU611895.1	23	12.56	37.1
OU611896.1	24	12.09	37.4
OU611897.1	25	10.88	37.3
OU611898.1	26	10.71	37.3
OU611899.1	27	8.6	37.9
OU611900.1	28	8.07	37.9
OU611901.1	29	7.56	38.6
OU611902.1	30	7.12	38.3
OU611873.1	W	18.66	39.9
OU611871.1	Z	20.78	37.3
OU611903.1	MT	0.02	18.9

The assembly has a BUSCO v5.3.2 ( Manni *et al.,* 2021) completeness of 99.2% (single 98.8%, duplicated 0.4%) using the lepidoptera_odb10 reference set (n=5,286). While not fully phased, the assembly deposited is of one haplotype. Contigs corresponding to the second haplotype have also been deposited.

## Genome annotation report

The ilEupTran1.1 genome has been annotated using the Ensembl BRAKER2 annotation pipeline ( Table 1; https://rapid.ensembl.org/Eupsilia_transversa_GCA_914767815.1/). The resulting annotation includes 18,267 transcribed mRNAs from 18,065 protein-coding genes.

## Methods

### Sample acquisition and nucleic acid extraction

A single female *E. transversa* specimen (ilEupTran1; genome assembly, Hi-C) was collected using a light trap from Wytham Woods, Berkshire, UK (latitude 51.774, longitude -1.331) by Liam Crowley (University of Oxford). The specimen was identified by Liam Crowley and snap-frozen on dry ice.

A single *E. transversa* specimen (ilEupTran2; RNA-Seq) of unknown sex was collected using a light trap from Lucas Road, High Wycombe, Buckinghamshire, UK (latitude 51.63, longitude -0.74) by David Lees (Natural History Museum). The specimen was identified by David Lees and dry frozen at -80 degrees.

DNA was extracted at the Tree of Life laboratory, Wellcome Sanger Institute. The ilEupTran1 sample was weighed and dissected on dry ice with tissue set aside for Hi-C sequencing. Thorax and abdomen tissue was cryogenically disrupted to a fine powder using a High molecular weight (HMW) DNA was extracted using the Qiagen MagAttract HMW DNA extraction kit. Low molecular weight DNA was removed from a 200-ng aliquot of extracted DNA using 0.8X AMpure XP purification kit prior to 10X Chromium sequencing; a minimum of 50 ng DNA was submitted for 10X sequencing. HMW DNA was sheared into an average fragment size between 12-20 kb in a Megaruptor 3 system with speed setting 30. Sheared DNA was purified by solid-phase reversible immobilisation using AMPure PB beads with a 1.8X ratio of beads to sample to remove the shorter fragments and concentrate the DNA sample. The concentration of the sheared and purified DNA was assessed using a Nanodrop spectrophotometer and Qubit Fluorometer and Qubit dsDNA High Sensitivity Assay kit. Fragment size distribution was evaluated by running the sample on the FemtoPulse system. Covaris cryoPREP Automated Dry Pulveriser, receiving multiple impacts. Fragment size analysis of 0.01-0.5 ng of DNA was then performed using an Agilent FemtoPulse.

RNA was extracted from thorax tissue of ilEupTran2 in the Tree of Life Laboratory at the WSI using TRIzol, according to the manufacturer’s instructions. RNA was then eluted in 50 μl RNAse-free water and its concentration RNA assessed using a Nanodrop spectrophotometer and Qubit Fluorometer using the Qubit RNA Broad-Range (BR) Assay kit. Analysis of the integrity of the RNA was done using Agilent RNA 6000 Pico Kit and Eukaryotic Total RNA assay.

### Sequencing

Pacific Biosciences HiFi circular consensus and 10X Genomics Chromium read cloud sequencing libraries were constructed according to the manufacturers’ instructions. Sequencing was performed by the Scientific Operations core at the Wellcome Sanger Institute on Pacific Biosciences SEQUEL II (HiFi), Illumina NovaSeq 6000 (10X) and Illumina HiSeq 4000 (RNA-Seq) instruments. Hi-C data were generated in the Tree of Life laboratory from head tissue of ilEupTran1 using the Arima v2 kit and sequenced on a NovaSeq 6000 instrument.

### Genome assembly

Assembly was carried out with Hifiasm ( Cheng *et al.,* 2021); haplotypic duplication was identified and removed with purge_dups ( Guan *et al.,* 2020). One round of polishing was performed by aligning 10X Genomics read data to the assembly with longranger align, calling variants with freebayes ( Garrison & Marth, 2012). The assembly was then scaffolded with Hi-C data ( Rao *et al.,* 2014) using SALSA2 ( Ghurye *et al.,* 2019). The assembly was checked for contamination as described previously ( Howe *et al.,* 2021). Manual curation was performed using HiGlass ( Kerpedjiev *et al.,* 2018) and Pretext. The mitochondrial genome was assembled using MitoHiFi ( Uliano-Silva *et al.,* 2021), which performs annotation using MitoFinder ( Allio *et al.,* 2020). The genome was analysed and BUSCO scores generated within the BlobToolKit environment ( Challis *et al.,* 2020). Table 3 contains a list of all software tool versions used, where appropriate.

**Table 3.** Software tools used.

Software tool	Version	Source
Hifiasm	0.15.3-r339	Cheng *et al.,* 2021
purge_dups	1.2.3	Guan *et al.,* 2020
SALSA2	2.2	Ghurye *et al.,* 2019
longranger align	2.2.2	https:// support.10xgenomics.com/ genome-exome/software/ pipelines/latest/advanced/ other-pipelines
freebayes	1.3.1-17-gaa2ace8	Garrison & Marth, 2012
MitoHiFi	2.0 singularity	Uliano-Silva *et al.,* 2021
HiGlass	1.11.6	Kerpedjiev *et al.,* 2018
PretextView	0.2.x	https://github.com/wtsi- hpag/PretextView
BlobToolKit	3.2.6	Challis *et al.,* 2020

### Genome annotation

The Ensembl gene annotation system ( Aken *et al.,* 2016) was used to generate annotation for the *Eupsilia transversa* assembly (GCA_914767815.1). Annotation was created primarily through alignment of transcriptomic data to the genome, with gap filling via protein-to-genome alignments of a select set of proteins from UniProt ( UniProt Consortium, 2019).

### Ethics/compliance issues

The materials that have contributed to this genome note have been supplied by a Darwin Tree of Life Partner. The submission of materials by a Darwin Tree of Life Partner is subject to the Darwin Tree of Life Project Sampling Code of Practice. By agreeing with and signing up to the Sampling Code of Practice, the Darwin Tree of Life Partner agrees they will meet the legal and ethical requirements and standards set out within this document in respect of all samples acquired for, and supplied to, the Darwin Tree of Life Project. Each transfer of samples is further undertaken according to a Research Collaboration Agreement or Material Transfer Agreement entered into by the Darwin Tree of Life Partner, Genome Research Limited (operating as the Wellcome Sanger Institute), and in some circumstances other Darwin Tree of Life collaborators.

## Data availability

European Nucleotide Archive: Eupsilia transversa (the satellite). Accession number PRJEB46318; https://identifiers.org/ena.embl/PRJEB46318.

The genome sequence is released openly for reuse. The *E. transversa* genome sequencing initiative is part of the Darwin Tree of Life (DToL) project. All raw sequence data and the assembly have been deposited in INSDC databases. Raw data and assembly accession identifiers are reported in Table 1.

## Author information

Members of the University of Oxford and Wytham Woods Genome Acquisition Lab are listed here: https://doi.org/10.5281/zenodo.6418202.

Members of the Natural History Museum Genome Acquisition Lab are listed here: https://doi.org/10.5281/zenodo.6418229.

Members of the Darwin Tree of Life Barcoding collective are listed here: https://doi.org/10.5281/zenodo.6418156.

Members of the Wellcome Sanger Institute Tree of Life programme are listed here: https://doi.org/10.5281/zenodo.6866293.

Members of Wellcome Sanger Institute Scientific Operations: DNA Pipelines collective are listed here: https://doi.org/10.5281/zenodo.5746904.

Members of the Tree of Life Core Informatics collective are listed here: https://doi.org/10.5281/zenodo.6125046.

Members of the Darwin Tree of Life Consortium are listed here: https://doi.org/10.5281/zenodo.6418363.

## References

[ref-1] Aken BL Ayling S Barrell D : The Ensembl Gene Annotation System. *Database (Oxford).* 2016;2016:baw093. 10.1093/database/baw093 27337980 PMC4919035

[ref-2] Allio R Schomaker-Bastos A Romiguier J : MitoFinder: Efficient Automated Large-Scale Extraction of Mitogenomic Data in Target Enrichment Phylogenomics. *Mol Ecol Resour.* 2020;20(4):892–905. 10.1111/1755-0998.13160 32243090 PMC7497042

[ref-3] Challis R Richards E Rajan J : BlobToolKit - Interactive Quality Assessment of Genome Assemblies. *G3 (Bethesda).* 2020;10(4):1361–74. 10.1534/g3.119.400908 32071071 PMC7144090

[ref-4] Cheng H Concepcion GT Feng X : Haplotype-Resolved *de Novo* Assembly Using Phased Assembly Graphs with Hifiasm. *Nat Methods.* 2021;18(2):170–75. 10.1038/s41592-020-01056-5 33526886 PMC7961889

[ref-5] Garrison E Marth G : Haplotype-Based Variant Detection from Short-Read Sequencing. arXiv: 1207.3907.2012. 10.48550/arXiv.1207.3907

[ref-6] Ghurye J Rhie A Walenz BP : Integrating Hi-C Links with Assembly Graphs for Chromosome-Scale Assembly. *PLoS Comput Biol.* 2019;15(8):e1007273. 10.1371/journal.pcbi.1007273 31433799 PMC6719893

[ref-7] Gordon : A List of the Macro-Lepi-Doptera of Wigtownshire. *Transactions and Journal of Proceedings of the Dumfriesshire and Galloway Natural History & Antiquarian Society.* 1913;3(1):168–88.

[ref-8] Guan D McCarthy SA Wood J : Identifying and Removing Haplotypic Duplication in Primary Genome Assemblies. *Bioinformatics.* 2020;36(9):2896–98. 10.1093/bioinformatics/btaa025 31971576 PMC7203741

[ref-9] Heath J Emmett AM : The Moths and Butterflies of Great Britain and Ireland, Volume 10: Noctuidae (Part II) and Agaristidae. Harley Books, Colchester,1983;10. Reference Source

[ref-10] Henwood B Sterling P : Field Guide to the Caterpillars of Great Britain and Ireland.Bloomsbury Publishing.2020. Reference Source

[ref-11] Howe K Chow W Collins J : Significantly Improving the Quality of Genome Assemblies through Curation. *GigaScience.* 2021;10(1):giaa153. 10.1093/gigascience/giaa153 33420778 PMC7794651

[ref-12] Kerpedjiev P Abdennur N Lekschas F : HiGlass: Web-Based Visual Exploration and Analysis of Genome Interaction Maps. *Genome Biol.* 2018;19(1):125. 10.1186/s13059-018-1486-1 30143029 PMC6109259

[ref-13] Manni M Berkeley MR Seppey M : BUSCO Update: Novel and Streamlined Workflows along with Broader and Deeper Phylogenetic Coverage for Scoring of Eukaryotic, Prokaryotic, and Viral Genomes. *Mol Biol Evol.* 2021;38(10):4647–54. 10.1093/molbev/msab199 34320186 PMC8476166

[ref-14] Rao SSP Huntley MH Durand NC : A 3D Map of the Human Genome at Kilobase Resolution Reveals Principles of Chromatin Looping. *Cell.* 2014;159(7):1665–80. 10.1016/j.cell.2014.11.021 25497547 PMC5635824

[ref-15] Uliano-Silva M Nunes JGF Krasheninnikova K : marcelauliano/MitoHiFi: mitohifi_v2.0. 2021. 10.5281/zenodo.5205678

[ref-16] UniProt Consortium: UniProt: A Worldwide Hub of Protein Knowledge. *Nucleic Acids Res.* 2019;47(D1):D506–15. 10.1093/nar/gky1049 30395287 PMC6323992

[ref-17] Waring P Townsend M : Field Guide to the Moths of Great Britain and Ireland: Third Edition.Bloomsbury Publishing,2017. Reference Source

