# Supplementary material for: The genome sequence of the satellite, Eupsilia transversa (Hufnagel, 1766)
Source: Wellcome Open Res. 2022 Oct 19;7:266. [Version 1] doi: 10.12688/wellcomeopenres.18105.1 (PMC11316177; doi:10.12688/wellcomeopenres.18105.1)
Supplement: Peer review history

# Reviewer report: Reviewer response for version 1

**DOI:** 10.21956/wellcomeopenres.20076.r86113

**Published:** 2024-08-09

**Authors:** Judith Risse

**Affiliations:** 1 Bioinformatics, Plant Sciences Group, Wageningen University & Research, Wageningen, Gelderland, The Netherlands; 2 KNAW-NIOO Netherlands , Wageningen University, Wageningen, The Netherlands

The paper is written concisely and adequately describes the genome assembly of the satellite moth.

**With regards to the rationale:**

- There is no mention of why this species was sequenced and assembled. A simple statement about the aim of DToL or Wytham Woods lab would suffice.

**With regards to the methods, I have a few questions, mostly for clarification:**

- What was the fragment size distribution for the 10X LMW fraction? Why was the LMW fraction chosen at all, 10X is normally done with HMW DNA?
- Was the RNAseq polyA selected? Strand specific? How many reads?
- Did you perform any adapter or quality trimming on any of the read sets? If so, which tools and parameters were used?
- Which coverage cutoff values for the different classes were used for purge_dups?
- Could you provide all parameters used in the analysis in Table 3 were they deviate from default? Alternatively, is there a link to the pipeline or scripts used in the analysis?

**Finally, a small textual error:**

- Missing end of sentence: "fine powder using a High molecular weight"
- Is this the remainder of the sentence?: Covaris cryoPREP ...

Are sufficient details of methods and materials provided to allow replication by others?

Partly

Is the rationale for creating the dataset(s) clearly described?

No

Are the datasets clearly presented in a useable and accessible format?

Yes

Are the protocols appropriate and is the work technically sound?

Yes

Reviewer Expertise:

Genomics and transcriptomics, genome assembly, population genomics,

I confirm that I have read this submission and believe that I have an appropriate level of expertise to confirm that it is of an acceptable scientific standard.

# Reviewer report: Reviewer response for version 1

**DOI:** 10.21956/wellcomeopenres.20076.r54006

**Published:** 2023-01-25

**Authors:** Katja Fischer

**Affiliations:** 1 QIMR Berghofer Medical Research Institute, Brisbane, Australia

This is a well written manuscript, however the rationale for creating the dataset is unclear. Why is it important to study the genome of this species?

 I understand there is no discussion in these reports, but a section outlining how this genome compares to others of related species would be useful.

Are sufficient details of methods and materials provided to allow replication by others?

Yes

Is the rationale for creating the dataset(s) clearly described?

No

Are the datasets clearly presented in a useable and accessible format?

Yes

Are the protocols appropriate and is the work technically sound?

Yes

Reviewer Expertise:

mites

I confirm that I have read this submission and believe that I have an appropriate level of expertise to confirm that it is of an acceptable scientific standard.
